# Characterization of dairy cattle production systems in two micro-watersheds of the Peruvian Amazonian Andes

**DOI:** 10.3389/fvets.2026.1936252

**Published:** 2026-10-14

**Authors:** Héctor V. Vásquez, Leidy G. Bobadilla, Leandro Valqui, Miguel A. Altamirano-Tantalean, Donna E. Ruiz, Milton J. Trigoso

**Affiliations:** 1 Laboratorio de Agrostología, Instituto de Investigación en Ganadería y Biotecnología, Facultad de Ingeniería Zootecnista, Biotecnología, Agronegocios y Ciencia de Datos, Universidad Nacional Toribio Rodríguez de Mendoza de Amazonas, Chachapoyas, Peru; 2 Escuela Profesional de Ingeniería en Agronegocios, Facultad de Ingeniería Zootecnista, Biotecnología, Agronegocios y Ciencia de Datos, Universidad Nacional Toribio Rodríguez de Mendoza de Amazonas, Chachapoyas, Peru; 3 Facultad de Ingeniería Zootecnista, Biotecnología, Agronegocios y Ciencia de Datos, Universidad Nacional Toribio Rodríguez de Mendoza de Amazonas, Chachapoyas, Peru

**Keywords:** dairy production systems, dairy value chain, farm typology, food security, Peruvian Amazon, small-scale producers

## Abstract

The Peruvian Andean Amazon is home to small-scale dairy livestock production that sustains numerous rural families, but whose heterogeneity complicates the design of targeted interventions. Characterizing and comparing production systems according to their similarities and differences represents an essential step to guide development policies. This study aimed to characterize and compare the dairy cattle production systems of two micro-watersheds in the Amazonas Region, Peru (Molinopampa and Pomacochas), to identify the patterns that differentiate their productive systems. A structured survey was administered to 621 producers, and the data were analyzed through comparison tests and multivariate analyses organized by thematic affinity. The results showed that the differences between the two micro-watersheds were primarily concentrated in milk marketing and in the breed composition of the herds, rather than in their production structure. The most pronounced contrast corresponded to milk price, which was higher in Pomacochas. Molinopampa was characterized by a higher proportion of female producers, greater use of concentrate feed and vaccination with both vaccines, a predominance of Brown Swiss cattle, and milk sales through associations or cooperatives, as well as direct sales or self-consumption. Pomacochas was distinguished by its integration with a processing company, a higher milk price, and higher perceived profitability. Self-reported mastitis was more frequent in Molinopampa, whereas piroplasmosis did not differ significantly between micro-watersheds. Both micro-watersheds shared a low deworming coverage, the perception of high operating costs, and limited coverage of technical assistance, which reached less than 13% of the producers. These findings show that both micro-watersheds may require territorially differentiated intervention strategies and provide a basis for designing more relevant dairy development policies in the Peruvian Andean Amazon.

## 1 Introduction

Cattle farming is one of the animal production systems of greatest socioeconomic importance worldwide (1). In 2024, global milk production reached 950 million tons (Mt), with a growth trend of 1.8% through 2034, the year it is expected to reach 1,146 Mt. (2), in a scenario that seeks to meet the demand of more than 6 billion consumers and sustain the livelihoods of 110 million dairy producers (3). In this way, the sector seeks to supply animal-source foods, generate income, and strengthen the productive assets of rural households, and in turn, contribute directly and indirectly to the Sustainable Development Goals (4).

In Peru, 5,842,166 cattle have been recorded, of which 16% are lactating cows; of this proportion, the Amazonas Region accounts for 81,403 lactating cows, which contribute 4.8% of national milk production (5). A large share of this population comes from livestock systems in the north of the Amazonas Region, in the provinces of Chachapoyas, Bongará, Rodríguez de Mendoza, and Utcubamba, localities where Fleckvieh cattle production systems have been documented (6). However, pastoral systems display a high diversity of breeds, with productive and structural variations along altitudinal gradients that modulate physiological response and milk composition (7); this breed and environmental heterogeneity makes the characterization of livestock systems indispensable to guide differentiated strategies of genetic improvement and productive management adjusted to the producer’s profile (8).

In the Amazonas Region, the Molinopampa and Pomacochas micro-watersheds constitute two key productive strongholds of the Region’s economy; both micro-watersheds are devoted to milk production, with a predominance of dairy cattle and improved breeds, and are located above 2,000 m a.s.l. (9), forming part of the ecoregion of the sub-Andean and Andean slopes of the eastern flank of the Peruvian Andes, also known as the yunga region (10). In these micro-watersheds, multiple problems persist that have led a large share of producers to offset yield losses by increasing stocking rates, which has undermined pasture availability and increased dependence on herd size (11, 12). Added to this is the impact of climate change on rainfall frequency, with projections of extreme drought (13) and the degradation of improved pastures (14). According to national statistics, this affects to a greater extent 41% of Peruvian agricultural households, who live in conditions of monetary poverty, nearly twice the rate observed in non-agricultural households (22%), with 98% of them managing family farming production units (15).

Along with climatic and productive pressures, animal health represents a critical threat to cattle farming. Bovine rabies, transmitted by *Desmodus rotundus*, and blackleg, caused by *Clostridium chauvoei*, directly affect the Region’s livestock. Peru is one of the countries with the highest incidence of bovine rabies in Latin America, with a range of 505–724 deaths per year, 465 outbreaks per million cattle, and estimated economic losses of US$121,797–171,992 (16, 17); in contrast, blackleg was declared by the Peruvian Ministry of Agrarian Development and Irrigation as a prevalent disease in the Amazonas Region, and mandatory annual vaccination campaigns were established after its designation as an enzootic zone (18). In this context, the characterization of vaccination coverage and health practices is essential to plan control strategies, given that access to adequate health interventions is often limited and requires responses tailored to the barriers of each production system (19).

Within this framework, farm-level characterization of livestock systems is an indispensable methodological tool to document the current situation of producers, the limitations they face, and the opportunities their production systems offer (20). This approach is grounded in the sustainable livelihoods framework (21), which recognizes that the trajectories of rural households result from the interaction among human, social, physical, financial, and natural assets, as well as the institutional and agroecological context in which they operate. By integrally addressing the sociodemographic, structural, productive, health, and commercial dimensions, farm-level characterization allows describing the heterogeneity of livestock systems and generating empirical evidence to design differentiated interventions, target public policies, and guide extension programs that respond to that real heterogeneity (22).

Despite the economic and social importance of the Molinopampa and Pomacochas micro-watersheds, the available information on their dairy livestock systems is fragmentary and does not allow comparison of the productive, health, and commercial conditions faced by their producers. In this regard, the sociodemographic, structural, productive, health, and commercial characterization of dairy producers in these micro-watersheds arises from the need to identify the patterns that characterize and differentiate dairy cattle producers in the Peruvian Andean Amazon. Therefore, the present study aimed to characterize the dairy livestock systems of the Molinopampa and Pomacochas micro-watersheds through the evaluation of sociodemographic conditions, herd structure, area and productive performance, breed composition of the herd, productive and health management, and economic and institutional linkages, as well as the perceived challenges of their producers. This characterization will help identify the main lines of governmental intervention, as well as the extension services and public investment required to promote practices that improve producers’ profitability and contribute to the development of a resilient and sustainable cattle farming sector.

## 2 Materials and methods

### 2.1 Study area

The study was carried out in the Molinopampa and Pomacochas micro-watersheds, two of the three main dairy production watersheds of the Amazonas Region (23), located in the provinces of Chachapoyas and Bongará, respectively (Figure 1). Both micro-watersheds record maximum temperatures of 25 to 29 °C and minimum temperatures of 11 to 17 °C, with high relative humidity and rainfall distributed throughout the year (24).

**Figure 1:**
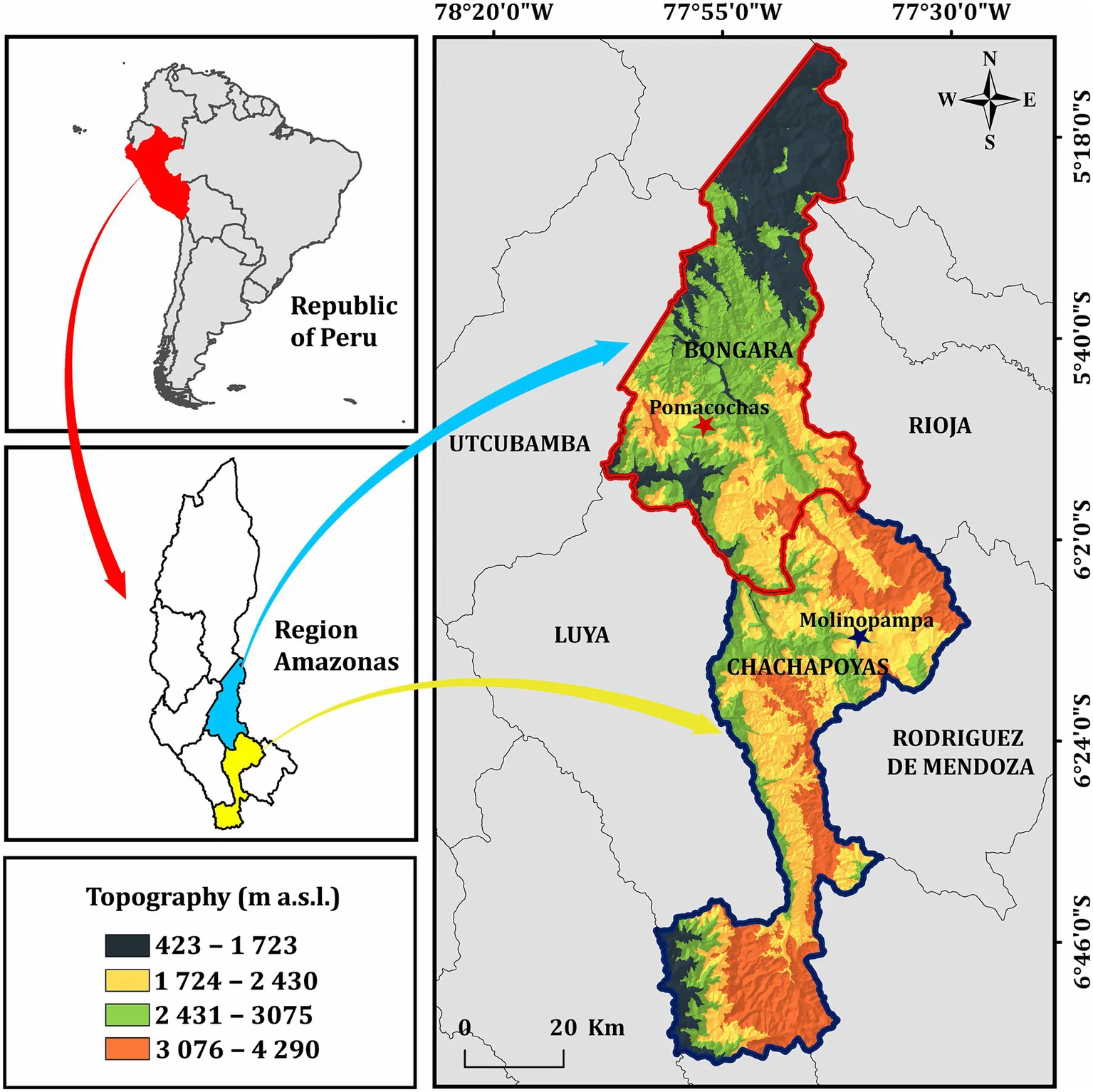
Location of the Molinopampa and Pomacochas micro-watersheds in the Amazonas Region.

### 2.2 Sampling and data collection

The unit of analysis was the dairy cattle producer, defined as the person responsible for the technical and economic management of a production unit with dairy cattle in production. The sampling frame comprised 1,569 producers registered before the study began, distributed between Molinopampa (*N*₁ = 778) and Pomacochas (*N*₂ = 791) (25). Stratified probability sampling was used, with each district considered an independent stratum. The minimum sample size was calculated separately for each stratum using the formula for finite populations (26) (Equation 1).


$$n=\frac{N\times Z^{2}\times p\times q}{e^{2}\times (N-1)+Z^{2}\times p\times q}$$ (1)

Where n is the minimum sample size required in each stratum, N is the population size of the stratum, *Z* = 1.96 is the value corresponding to a 95% confidence level, *p* = 0.50 is the expected proportion, *q* = 1 − *p* = 0.50 is the complementary proportion, and *e* = 0.05 is the allowable margin of error. Applying the formula yielded minimum sample sizes of 257.39 producers for Molinopampa and 258.80 for Pomacochas. To compensate for an anticipated nonresponse rate of 20%, these sample sizes were adjusted using Equation 2:


$$n_{\text{adjusted}}=\frac{n}{1–r\;}$$ (2)

Where *r* = 0.20 represents the expected nonresponse rate. After the adjustment, a planned sample of 321 producers in Molinopampa and 324 in Pomacochas was established, for a total of 645 producers. Within each stratum, the producers in the sampling frame were individually identified, and participants were selected by simple random sampling without replacement using random numbers generated in Microsoft Excel. Thus, each eligible producer included in the sampling frame had a known probability of being selected within their respective stratum. Nonresponse cases consisted of selected producers who declined to participate, could not be contacted, or submitted incomplete questionnaires; these cases were not replaced. Data were collected between January and April 2025 through the in-person administration of a structured questionnaire. After fieldwork was completed and the information had undergone quality control, 310 valid questionnaires were obtained in Molinopampa and 311 in Pomacochas, yielding an effective analytical sample of 621 producers. Relative to the planned sample, the proportion of valid questionnaires was 96.6% in Molinopampa and 96.0% in Pomacochas. Despite nonresponse and the exclusion of incomplete questionnaires, the final number of valid surveys in both strata remained above the minimum sample size initially required.

The surveys were administered according to the following inclusion criteria: (i) being a producer with at least one lactating cow under their technical and economic management, (ii) residing permanently in the micro-watersheds studied, (iii) being over 18 years of age, (iv) being directly responsible for herd management, (v) having at least one year of experience, and (vi) providing informed consent to participate voluntarily. The surveys were designed exclusively for dairy cattle producers. To validate the instrument, a pilot test was conducted with 8 producers per micro-watershed before large-scale data collection; these data were not included in the present study. The survey was divided into 7 dimensions: sociodemographic profile, herd structure, land area and production performance, herd breed composition, production and health management, economic and institutional ties, and challenges perceived by producers, as shown in Table 1. The complete questionnaire used for data collection is provided as Supplementary Material S1.

**Table 1:** Quantitative and qualitative variables used for the characterization of dairy cattle production systems.

Type	Dimension	Variables
Quantitative	Sociodemographic profile	Experience as a livestock producer (years).
	Herd structure	Number of lactating cows; number of male or female calves; number of breeding bulls.
	Area and productive performance	Total farm area per producer (ha); daily milk production per herd (L day^−1^); milk sale price (PEN L^−1^).
Qualitative	Sociodemographic profile	Sex (female, male); educational level (no education, primary, secondary, technical, university).
	Breed composition of the herd	Breeds present in the herd (Brown Swiss, Fleckvieh, Simmental, Holstein, Jersey, Criollo); herd breed typology (single-breed, two-breed, multi-breed) (Supplementary Figure S1); type of reproduction (natural mating, artificial insemination, both).
	Productive and health management	Feeding system (rotational grazing, continuous grazing, cut-and-carry); type of supplementation (none, mineral salts, concentrate, salts and concentrates); vaccination plan (none, at least one [blackleg or rabies], both vaccines applied); deworming (yes, no); self-reported occurrence of clinical mastitis during the previous year (yes, no); self-reported occurrence of piroplasmosis during the previous year (yes, no).
	Economic and institutional linkages	Sales channel (association or cooperative, processing company, direct sale or self-consumption); perception of profitability (profitable, not profitable); source of technical assistance (local municipality, regional government, association or cooperative, SENASA (Servicio Nacional de Sanidad Agraria), multiple sources, none); access to credit (yes, no).
	Perceived challenges	Lack of pasture or forage; adverse climatic conditions; unstable market price; high operating costs; inadequate infrastructure; labor shortage (all: yes, no).

### 2.3 Data analysis

Data were analyzed using R version 4.5.0 (27) in the RStudio environment (28). The normality of quantitative variables was assessed using the Anderson–Darling test. Because the quantitative data were not normally distributed, the Mann–Whitney U test was used to compare the micro-watersheds. Cliff’s *δ* was also calculated as an effect size and interpreted according to the thresholds proposed by Romano et al. (29): negligible (<0.147), small (0.147 ≤ |*δ*| < 0.330), medium (0.330 ≤ |*δ*| < 0.474), and large (≥0.474). Categorical variables were analyzed using contingency tables and Pearson’s chi-square test; Fisher’s exact test was used when any expected cell frequency was less than 5. Cramér’s *V* was reported and classified according to the thresholds proposed by Kim (30), adjusted for the degrees of freedom of each table. The thresholds for small, medium, and large associations were 0.10, 0.30, and 0.50, respectively, and decreased progressively for tables with more categories. Odds ratios (ORs) with 95% confidence intervals were also calculated for binary variables (31), using Molinopampa as the reference micro-watershed and the first listed category of each binary variable as the reference category. For variables with more than two categories, adjusted residuals were calculated using a critical threshold of |*z*| > 2.58 (32).

To control the false discovery rate (FDR) associated with multiple comparisons, all *p*-values from the bivariate comparisons between micro-watersheds were treated as a single family of tests and adjusted together using the Benjamini–Hochberg procedure (33). The *p*-values reported in the tables are adjusted values. Spearman’s correlation coefficients were also calculated among the quantitative structural and production variables as a confirmatory check of the associations underlying the multivariate analysis. Correlations were interpreted according to Papageorgiou (34) as very weak (<0.20), weak (0.20–0.39), moderate (0.40–0.59), strong (0.60–0.79), or very strong (>0.80). Finally, principal component analysis (PCA) and three multiple correspondence analyses (MCAs) were performed. Quantitative variables were standardized before PCA. In the MCA, micro-watershed was included as a supplementary qualitative variable, with Molinopampa and Pomacochas as its categories. Separation between the two categories was quantified as the Euclidean distance between their coordinates on the first two dimensions of the factor plane. Effect sizes were calculated using the DescTools package (35), and multivariate analyses were performed using FactoMineR (36) and factoextra (37). For the bivariate comparisons, statistical significance was defined as an adjusted *p*-value < 0.05.

## 3 Results

### 3.1 Quantitative variables of the systems

The results revealed that both micro-watersheds differed significantly in six of the quantitative variables evaluated, with the only exception being experience as a livestock producer (19.7 ± 12.9 vs. 19.2 ± 12.8 years; *p* = 0.707; *δ* = −0.021, negligible). Pomacochas showed significantly higher values than Molinopampa in the number of lactating cows (8.0 ± 5.7 vs. 6.4 ± 4.1; *p* = 0.008; *δ* = −0.147, small), number of female or male calves (7.2 ± 5.1 vs. 6.9 ± 6.1; *p* = 0.010; *δ* = −0.148, small), number of breeding bulls (3.0 ± 3.7 vs. 2.3 ± 2.5; *p* = 0.008; *δ* = −0.129, negligible), total farm area per producer (17.2 ± 15.0 vs. 13.6 ± 11.9 ha; *p* < 0.001; *δ* = −0.176, small), milk yield per herd (48.0 ± 41.2 vs. 41.8 ± 36.8 L day^−1^; *p* = 0.042; *δ* = −0.103, negligible), and milk price (1.67 ± 0.30 vs. 1.52 ± 0.18 PEN L^−1^; *p* < 0.001; *δ* = −0.488, large). Although statistically significant, the differences in the number of breeding bulls and milk yield were negligible in magnitude, whereas those associated with the number of lactating cows, number of female or male calves, and total farm area per producer were small. Overall, milk price was the only variable with a large effect size, representing the contrast of greatest magnitude between micro-watersheds (Table 2).

**Table 2:** Comparison of quantitative variables between the livestock micro-watersheds.

Variable	Molinopampa		Pomacochas		*p*-value	Cliff’s *δ*	Magnitude
	Mean ± SD	Median [Q1–Q3]	Mean ± SD	Median [Q1–Q3]			
Producer profile							
Experience as a livestock producer (years)	19.2 ± 12.8 a	15 [10–28]	19.7 ± 12.9 a	18 [10–25]	0.707	−0.021	Negligible
Herd and farm structure							
Number of lactating cows	6.4 ± 4.1 b	6 [4–8]	8.0 ± 5.7 a	7 [4–10]	0.008	−0.147	Small
Number of female or male calves per herd	6.9 ± 6.1 b	5 [4–8]	7.2 ± 5.1 a	6 [4–10]	0.01	−0.148	Small
Number of breeding bulls per herd	2.3 ± 2.5 b	2 [1–3]	3.0 ± 3.7 a	2 [1–4]	0.008	−0.129	Negligible
Total farm area per producer (ha)	13.6 ± 11.9 b	10 [5–18]	17.2 ± 15.0 a	12 [8–20]	<0.001	−0.176	Small
Productive and economic performance							
Milk yield (L day^−1^)	41.8 ± 36.8 b	30 [18–50]	48.0 ± 41.2 a	35 [21–60]	0.042	−0.103	Negligible
Milk price (PEN L^−1^)	1.52 ± 0.18 b	1.50 [1.50–1.50]	1.67 ± 0.30 a	1.70 [1.50–1.70]	<0.001	−0.488	Large

Values are presented as mean ± standard deviation. Different letters in the same row indicate significant differences between micro-watersheds according to the Mann–Whitney *U* test (*p* < 0.05). Effect size classification: negligible (|*δ*| < 0.147), small (0.147 ≤ |*δ*| < 0.33), medium (0.33 ≤ |*δ*| < 0.474), and large (|*δ*| ≥ 0.474). PEN = Peruvian sol.

The principal component analysis (PCA) explained 52.6% of the total variance in the first two dimensions (Dim. 1 = 37.0%; Dim. 2 = 15.6%; Figure 2A). In Dim. 1, the variable with the greatest contribution was the number of lactating cows (26.9%), and in Dim. 2, the milk price (56.6%). The biplot showed that the ellipses of both micro-watersheds widely overlapped and their centroids were located close to the origin of the factor plane. Although the univariate variables differed between micro-watersheds (Table 2), the PCA did not discriminate the systems as separate groups in the factorial space.

**Figure 2:**
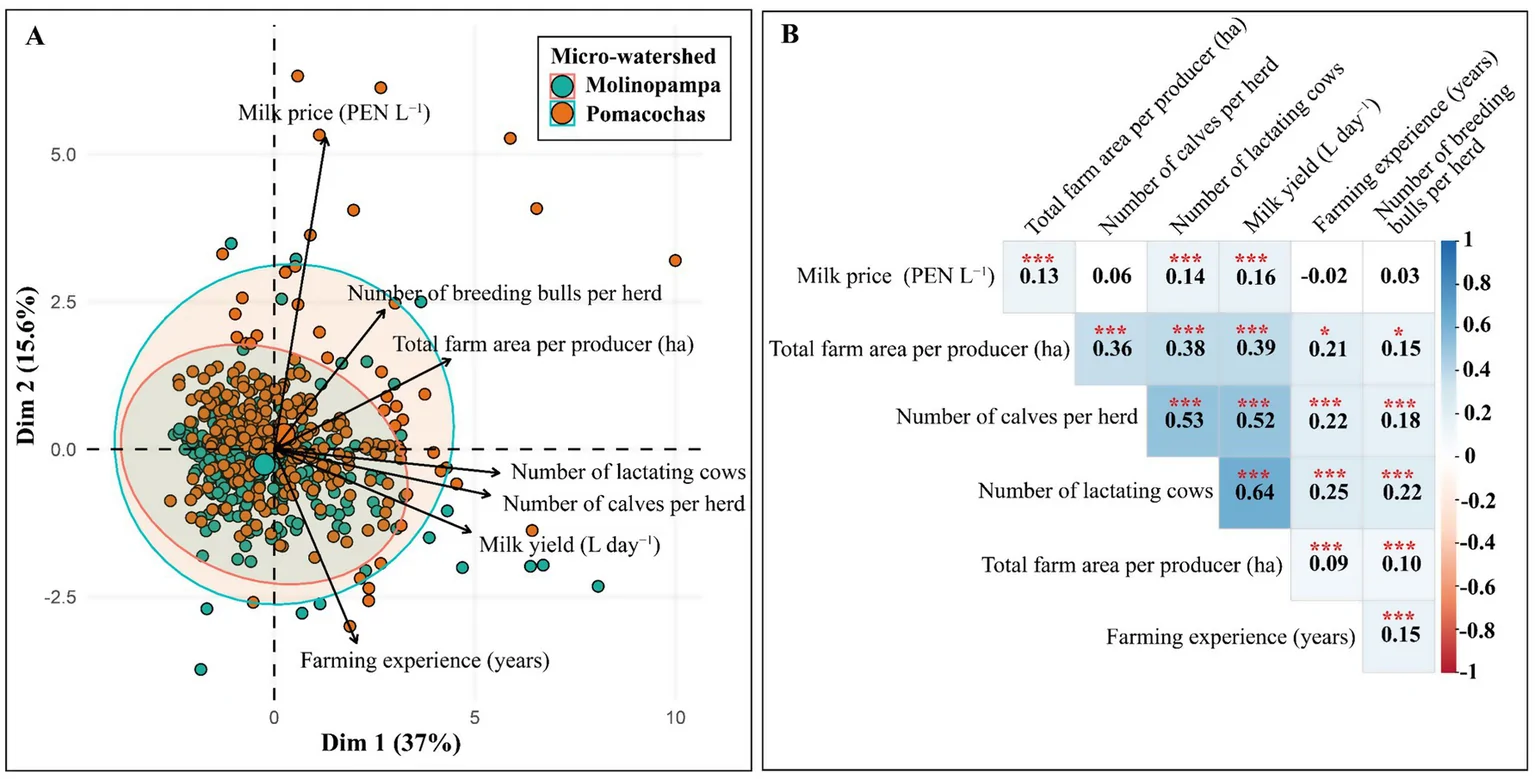
General structural analysis of both livestock micro-watersheds. Principal component analysis **(A)** and Spearman’s correlation **(B)**.

The vectors of the number of lactating cows, milk yield per herd, and number of female or male calves pointed in the same direction and formed acute angles with each other. This pattern was confirmed by Spearman’s correlation analysis (Figure 2B), where the number of lactating cows and milk yield per herd showed the most marked association (*r* = 0.64; *p* < 0.001), classified as strong, followed by the number of female or male calves with the number of lactating cows (*r* = 0.53; *p* < 0.001) and with milk yield per herd (*r* = 0.52; *p* < 0.001), both classified as moderate. Total farm area per producer was weakly associated with the number of female or male calves per herd (*r* = 0.36; *p* < 0.001), the number of lactating cows (*r* = 0.38; *p* < 0.001), and milk yield per herd (*r* = 0.39; *p* < 0.001). Milk price was very weakly associated with the number of lactating cows (*r* = 0.14; *p* < 0.001), milk yield per herd (*r* = 0.16; *p* < 0.001), and total farm area per producer (*r* = 0.13; *p* = 0.001), and showed no significant association with experience as a livestock producer, the number of female or male calves, or the number of breeding bulls (*p* > 0.05 in all three cases). Experience as a livestock producer showed very weak to weak associations with the other system variables (*r* = 0.03–0.25).

### 3.2 Sociodemographic characteristics

The sociodemographic composition of the producers differed between the Molinopampa and Pomacochas micro-watersheds in sex distribution and educational level. The sex distribution of producers showed significant differences between both micro-watersheds (*χ*^2^ = 26.98; *p* < 0.001; *V* = 0.208, small), with women representing 41.6% of the producers in Molinopampa compared with 22.2% in Pomacochas (OR = 2.50; 95% CI: 1.76–3.55). Educational level also differed between micro-watersheds (*χ*^2^ = 18.80; *p* = 0.002; *V* = 0.174, small). Adjusted residuals showed that no formal education was overrepresented in Molinopampa (3.5% vs. 0.0%; *z* = 3.35), whereas the secondary level was underrepresented in Molinopampa (45.8% vs. 54.7%; *z* = −2.21). The primary, technical, and university levels showed no significant deviations between micro-watersheds (Table 3).

**Table 3:** Sociodemographic characteristics in both livestock micro-watersheds.

Category	Molinopampa		Pomacochas		*χ* ^2^	*p*-value	Cramér’s *V*	OR [95% CI]	Adjusted *z*-residual
	n	%	n	%					
Sex					26.98	<0.001	0.208	2.50 [1.76–3.55]	–
Female	129	41.6	69	22.2	–	–	–	–	–
Male	181	58.4	242	77.8	–	–	–	–	–
Educational level					18.80	0.002	0.174	–	–
None	11	3.5	0	0.0	–	–	–	–	3.35
Primary	131	42.3	120	38.6	–	–	–	–	0.93
Secondary	142	45.8	170	54.7	–	–	–	–	−2.21
Technical	6	1.9	11	3.5	–	–	–	–	−1.22
University	20	6.5	10	3.2	–	–	–	–	1.88

The symbol (–) indicates that the statistic does not apply to that row.

### 3.3 Breed composition of the herd

Herd breed composition differed significantly between micro-watersheds for five of the six breeds evaluated (Table 4). The most marked contrasts corresponded to the Brown Swiss breed (*χ*^2^ = 115.47; *p* < 0.001; *V* = 0.431, medium) and the Fleckvieh breed (*χ*^2^ = 30.58; *p* < 0.001; *V* = 0.222, small). The Brown Swiss breed was present in 75.2% of the herds in Molinopampa compared with 32.2% of those in Pomacochas (OR = 0.16; 95% CI: 0.11–0.22), whereas the Fleckvieh breed showed an inverse pattern, with presence in 17.0% of the herds in Pomacochas compared with 3.5% of those in Molinopampa (OR = 5.58; 95% CI: 2.86–10.92). The other three breeds (Holstein, Jersey, and Simmental) also showed significant differences of small magnitude. Holstein and Jersey were more frequent in Molinopampa than in Pomacochas (21.0% vs. 12.2%, OR = 0.52; 95% CI: 0.34–0.81; and 8.1% vs. 1.9%, OR = 0.22; 95% CI: 0.09–0.55, respectively), whereas Simmental predominated in Pomacochas (37.9% vs. 23.5%; OR = 1.98; 95% CI: 1.40–2.81). The Criollo breed was the only one without significant differences between micro-watersheds (*χ*^2^ = 0.40; *p* = 0.600; *V* = 0.025), with similar presence in Molinopampa (12.9%) and Pomacochas (11.3%).

**Table 4:** Breed composition and typology of the herd per producer.

Category	Molinopampa		Pomacochas		*χ* ^2^	*p*-value	Cramér’s *V*	OR [95% CI]	Adjusted *z*-residual
	*n*	%	*n*	%					
Holstein					8.59	0.007	0.118	0.52 [0.34–0.81]	–
No	245	79.0	273	87.8	–	–	–	–	–
Yes	65	21.0	38	12.2	–	–	–	–	–
Brown Swiss					115.47	<0.001	0.431	0.16 [0.11–0.22]	–
No	77	24.8	211	67.8	–	–	–	–	–
Yes	233	75.2	100	32.2	–	–	–	–	–
Simmental					15.10	<0.001	0.156	1.98 [1.40–2.81]	–
No	237	76.5	193	62.1	–	–	–	–	–
Yes	73	23.5	118	37.9	–	–	–	–	–
Jersey					12.32	0.001	0.141	0.22 [0.09–0.55]	–
No	285	91.9	305	98.1	–	–	–	–	–
Yes	25	8.1	6	1.9	–	–	–	–	–
Criollo					0.40	0.600	0.025	0.86 [0.53–1.39]	–
No	270	87.1	276	88.7	–	–	–	–	–
Yes	40	12.9	35	11.3	–	–	–	–	–
Fleckvieh					30.58	<0.001	0.222	5.58 [2.86–10.92]	–
No	299	96.5	258	83.0	–	–	–	–	–
Yes	11	3.5	53	17.0	–	–	–	–	–
Breed typology					56.95	<0.001	0.303	–	–
Single-breed	194	62.6	275	88.4	–	–	–	–	−7.49
Two-breed	98	31.6	33	10.6	–	–	–	–	6.41
Multi-breed	18	5.8	3	1.0	–	–	–	–	3.34

The symbol (–) indicates that the statistic does not apply to that row.

The breed typology of the herd also differed between micro-watersheds (*χ*^2^ = 56.95; *p* < 0.001; *V* = 0.303, medium). Adjusted residuals indicated that Pomacochas had a higher proportion of single-breed herds (88.4% vs. 62.6% in Molinopampa; *z* = −7.49), whereas Molinopampa presented higher proportions of two-breed (31.6% vs. 10.6%; *z* = 6.41) and multi-breed herds (5.8% vs. 1.0%; *z* = 3.34).

### 3.4 Productive and health management

The forage provision systems and deworming did not show significant differences between micro-watersheds (*p* > 0.05). Rotational grazing was the predominant system in both (84.8% in Molinopampa and 83.0% in Pomacochas), followed by continuous grazing (9.7% vs. 14.1%) and cut-and-carry (5.5% vs. 2.9%); the differences did not reach statistical significance (*χ*^2^ = 5.16; *p* = 0.110; *V* = 0.091). Similarly, most producers did not deworm their cattle in both micro-watersheds (86.8% in Molinopampa and 91.3% in Pomacochas; *χ*^2^ = 3.29; *p* = 0.105; *V* = 0.073).

The most marked contrast in productive management corresponded to the type of supplementation (*χ*^2^ = 62.36; *p* < 0.001; *V* = 0.317, medium). Adjusted residuals showed that in Pomacochas the predominant producers were those who did not supplement (61.7% vs. 37.4% in Molinopampa; *z* = −6.06), whereas in Molinopampa the exclusive use of concentrate was more common (36.8% vs. 10.9%; *z* = 7.56). The type of reproduction (*χ*^2^ = 8.72; *p* = 0.022; *V* = 0.119, small) and the vaccination plan (*χ*^2^ = 7.32; *p* = 0.042; *V* = 0.109, small) also differed significantly, with associations of small magnitude. Artificial insemination was more frequent in Molinopampa (16.5% vs. 10.3%; *z* = 2.26) and natural mating in Pomacochas (72.0% vs. 61.3%; *z* = −2.84); the dual vaccination scheme against rabies and blackleg predominated in Molinopampa (41.6% vs. 31.2%; *z* = 2.70), whereas the application of a single vaccine was more frequent in Pomacochas (48.9% vs. 41.0%; *z* = −1.98).

Regarding animal health, the self-reported occurrence of clinical mastitis was higher in Molinopampa (29.4%) than in Pomacochas (17.0%) (*χ*^2^ = 13.22; *p* < 0.001; *V* = 0.146, small; OR = 0.49; 95% CI: 0.34–0.73). The self-reported occurrence of piroplasmosis did not differ significantly between micro-watersheds (*p* = 0.057; *V* = 0.085), with values of 18.1% in Molinopampa and 25.1% in Pomacochas (Table 5).

**Table 5:** Productive and health characteristics in both livestock micro-watersheds.

Category	Molinopampa		Pomacochas		*χ* ^2^	*p*-value	Cramér’s *V*	OR [95% CI]	Adjusted *z*-residual
	n	%	n	%					
Forage provision systems					5.16	0.110	0.091	–	–
Continuous grazing	30	9.7	44	14.1	–	–	–	–	−1.72
Rotational grazing	263	84.8	258	83.0	–	–	–	–	0.64
Cut-and-carry	17	5.5	9	2.9	–	–	–	–	1.61
Type of supplementation					62.36	<0.001	0.317	–	–
None	116	37.4	192	61.7	–	–	–	–	−6.06
Mineral salt only	48	15.5	54	17.4	–	–	–	–	−0.63
Concentrate only	114	36.8	34	10.9	–	–	–	–	7.56
Both	32	10.3	31	10.0	–	–	–	–	0.15
Type of reproduction					8.72	0.022	0.119	–	–
Natural mating	190	61.3	224	72.0	–	–	–	–	−2.84
Artificial insemination	51	16.5	32	10.3	–	–	–	–	2.26
Both	69	22.3	55	17.7	–	–	–	–	1.43
Vaccination plan					7.32	0.042	0.109	–	–
No vaccination	54	17.4	62	19.9	–	–	–	–	−0.80
One vaccine	127	41.0	152	48.9	–	–	–	–	−1.98
Both (rabies + blackleg)	129	41.6	97	31.2	–	–	–	–	2.70
Deworming					3.29	0.105	0.073	0.62 [0.37–1.04]	–
No deworming	269	86.8	284	91.3	–	–	–	–	–
Yes, deworming	41	13.2	27	8.7	–	–	–	–	–
Clinical mastitis					13.22	<0.001	0.146	0.49 [0.34–0.73]	–
No	219	70.6	258	83.0	–	–	–	–	–
Yes	91	29.4	53	17.0	–	–	–	–	–
Piroplasmosis					4.52	0.057	0.085	1.52 [1.00–2.24]	–
No	254	81.9	233	74.9	–	–	–	–	–
Yes	56	18.1	78	25.1	–	–	–	–	–

The symbol (–) indicates that the statistic does not apply to that row.

### 3.5 Economic linkages

The sales channel was the variable with the most marked contrast between micro-watersheds (*χ*^2^ = 112.69; *p* < 0.001; *V* = 0.426, medium). Adjusted residuals indicated that in Molinopampa the sale to associations or cooperatives (46.8% vs. 19.9% in Pomacochas; *z* = 7.09) and direct sale or self-consumption (37.7% vs. 24.4%; *z* = 3.58) predominated, whereas in Pomacochas the sale to processing companies dominated (55.6% vs. 15.5%; *z* = −10.45); significant deviations were observed in all three categories (|*z*| > 2.58). Perceived profitability also differed between micro-watersheds (*χ*^2^ = 26.90; *p* < 0.001; *V* = 0.208, small). Pomacochas had a higher proportion of producers who perceived their activity as profitable (88.7% vs. 72.3% in Molinopampa; OR = 3.03; 95% CI: 1.97–4.66). In contrast, the source of technical assistance did not differ significantly between micro-watersheds (*p* = 0.384; *V* = 0.098). In both micro-watersheds, most producers did not receive technical assistance (87.7% in Molinopampa and 92.9% in Pomacochas). None of the surveyed producers reported access to credit in either micro-watershed (0% in both cases); therefore, this variable was excluded from the inferential and multivariate analyses because it showed no variability. Overall, the micro-watersheds differed in sales channel and perceived profitability, whereas the lack of technical assistance was common in both (Table 6).

**Table 6:** Economic linkages in both livestock micro-watersheds.

Category	Molinopampa		Pomacochas		*χ* ^2^	*p*-value	Cramér’s *V*	OR [95% CI]	Adjusted *z*-residual
	*n*	%	*n*	%					
Sales channel					112.69	<0.001	0.426	–	–
Association/Cooperative	145	46.8	62	19.9	–	–	–	–	7.09
Processing company	48	15.5	173	55.6	–	–	–	–	−10.45
Direct sale/Self-consumption	117	37.7	76	24.4	–	–	–	–	3.58
Source of technical assistance					–	0.384	0.098	–	–
Local municipality	12	3.9	9	2.9	–	–	–	–	0.67
Regional government	9	2.9	5	1.6	–	–	–	–	1.09
Producers’ association	12	3.9	5	1.6	–	–	–	–	1.73
SENASA	2	0.6	2	0.6	–	–	–	–	0.00
Multiple sources	3	1.0	1	0.3	–	–	–	–	1.01
None received	272	87.7	289	92.9	–	–	–	–	−2.19
Perceived profitability					26.90	<0.001	0.208	3.03 [1.97–4.66]	–
Not profitable	86	27.7	35	11.3	–	–	–	–	–
Profitable	224	72.3	276	88.7	–	–	–	–	–

The symbol (–) indicates that the statistic does not apply to that row.

### 3.6 Perceived challenges

The perception of production challenges differed between micro-watersheds in three of the six categories evaluated. The six challenges can be grouped into two conceptual dimensions according to their nature: operational challenges (lack of forage, adverse climatic conditions, and labor shortage), related to productive and health management, and commercial challenges (unstable market prices, high operating costs, and inadequate infrastructure), related to the economic and institutional linkages of the production system. Within the operational challenges, the lack of forage was perceived by 51.6% of the producers in Molinopampa and 40.2% in Pomacochas (*χ*^2^ = 8.15; *p* = 0.009; *V* = 0.115, small; OR = 0.63; 95% CI: 0.46–0.87), whereas adverse climatic conditions were perceived by 65.5% of the producers in Molinopampa and 49.8% in Pomacochas (*χ*^2^ = 15.56; *p* < 0.001; *V* = 0.158, small; OR = 0.52; 95% CI: 0.38–0.72). Conversely, labor shortage was more frequent in Pomacochas (47.6%) than in Molinopampa (33.9%) (*χ*^2^ = 12.10; *p* = 0.001; *V* = 0.140, small; OR = 1.77; 95% CI: 1.28–2.45). On the other hand, the commercial challenges, unstable market prices, high operating costs, and inadequate infrastructure, did not show significant differences between micro-watersheds (*p* > 0.05) (Table 7).

**Table 7:** Perceived challenges of livestock producers in both micro-watersheds.

Category	Molinopampa		Pomacochas		*χ* ^2^	*p*-value	Cramér’s *V*	OR [95% CI]
	*n*	%	*n*	%				
Lack of forage					8.15	0.009	0.115	0.63 [0.46–0.87]
No	150	48.4	186	59.8	–	–	–	–
Yes	160	51.6	125	40.2	–	–	–	–
Adverse climatic conditions					15.56	<0.001	0.158	0.52 [0.38–0.72]
No	107	34.5	156	50.2	–	–	–	–
Yes	203	65.5	155	49.8	–	–	–	–
Unstable market price					3.95	0.074	0.080	0.72 [0.52–1.00]
No	169	54.5	194	62.4	–	–	–	–
Yes	141	45.5	117	37.6	–	–	–	–
High operating costs					0.002	0.985	0.002	1.01 [0.73–1.38]
No	163	52.6	163	52.4	–	–	–	–
Yes	147	47.4	148	47.6	–	–	–	–
Inadequate infrastructure					3.88	0.075	0.079	1.37 [1.00–1.88]
No	174	56.1	150	48.2	–	–	–	–
Yes	136	43.9	161	51.8	–	–	–	–
Labor shortage					12.10	0.001	0.140	1.77 [1.28–2.45]
No	205	66.1	163	52.4	–	–	–	–
Yes	105	33.9	148	47.6	–	–	–	–

The symbol (–) indicates that the statistic does not apply to that row.

### 3.7 Multivariate analysis of qualitative dimensions

#### 3.7.1 Producer profile

The multiple correspondence analysis of the four producer profile variables (sex, educational level, herd breed typology, and type of reproduction) yielded a cumulative percentage of 28.3% in its first two dimensions (Dim. 1 = 15.2%; Dim. 2 = 13.1%; Figure 3). Producers with a higher educational level, greater breed diversity and artificial insemination were positioned at one end, associated with Molinopampa, whereas producers with natural mating and single-breed herds were located at the opposite end, associated with Pomacochas. The distance between the two micro-watersheds was 0.559.

**Figure 3:**
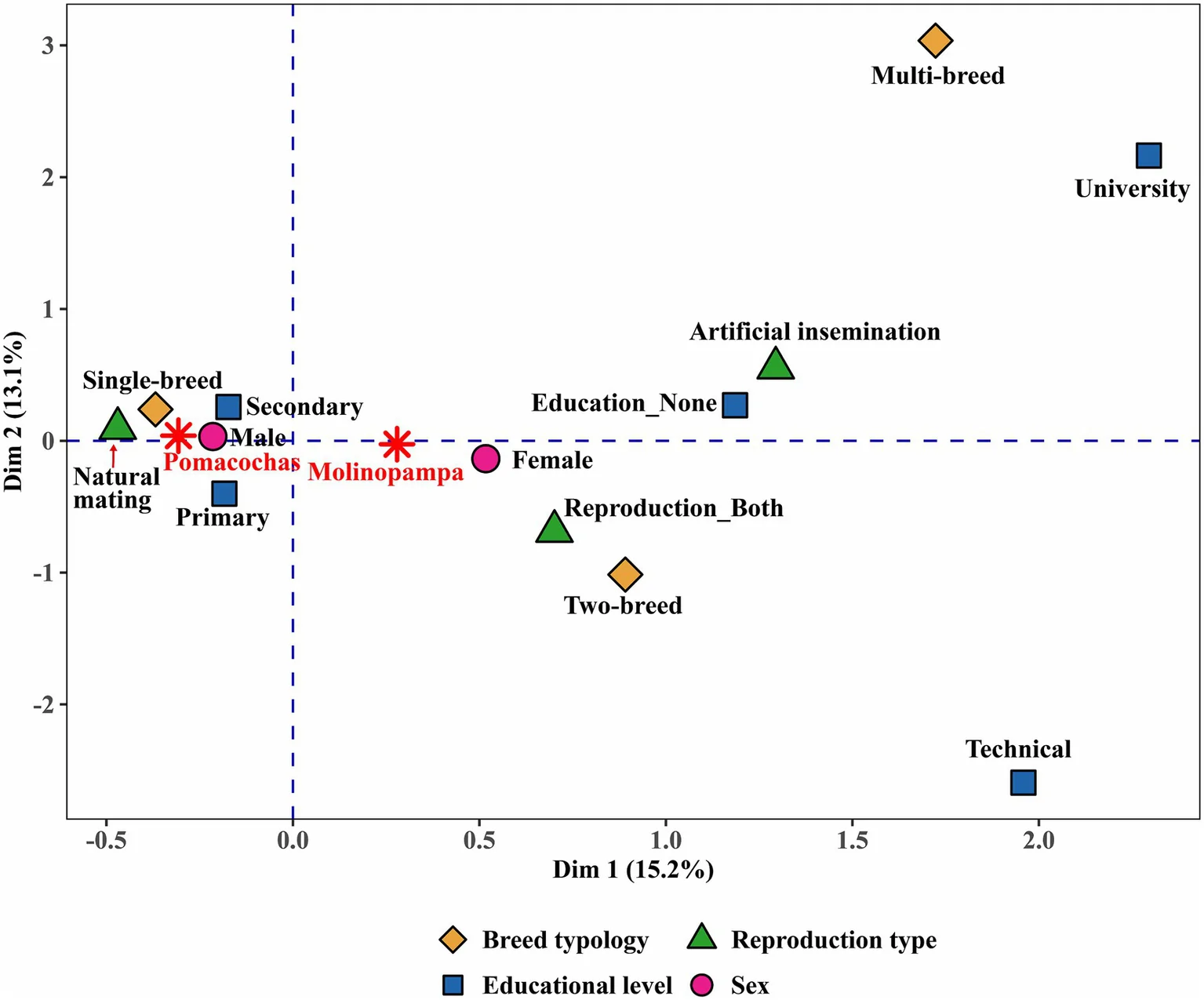
Multiple correspondence analysis of the producer profile.

#### 3.7.2 Productive-health management and operational challenges

The multiple correspondence analysis of the nine variables related to management, animal health, and operational challenges (feeding modality, type of supplementation, vaccination plan, deworming, mastitis, piroplasmosis, lack of forage, adverse climatic conditions, and labor shortage) indicated a cumulative percentage of 26.3% in its first two dimensions (Dim. 1 = 14.4%; Dim. 2 = 11.9%; Figure 4). Management practices such as deworming, the use of concentrate, and cut-and-carry feeding, were positioned at one end, associated with Molinopampa, whereas continuous grazing and the absence of supplementation were located at the opposite end, associated with Pomacochas. The distance between the two micro-watersheds was 0.550. Mastitis and piroplasmosis were positioned on opposite sides of the factorial plane; however, piroplasmosis did not differ significantly between micro-watersheds in the bivariate analysis.

**Figure 4:**
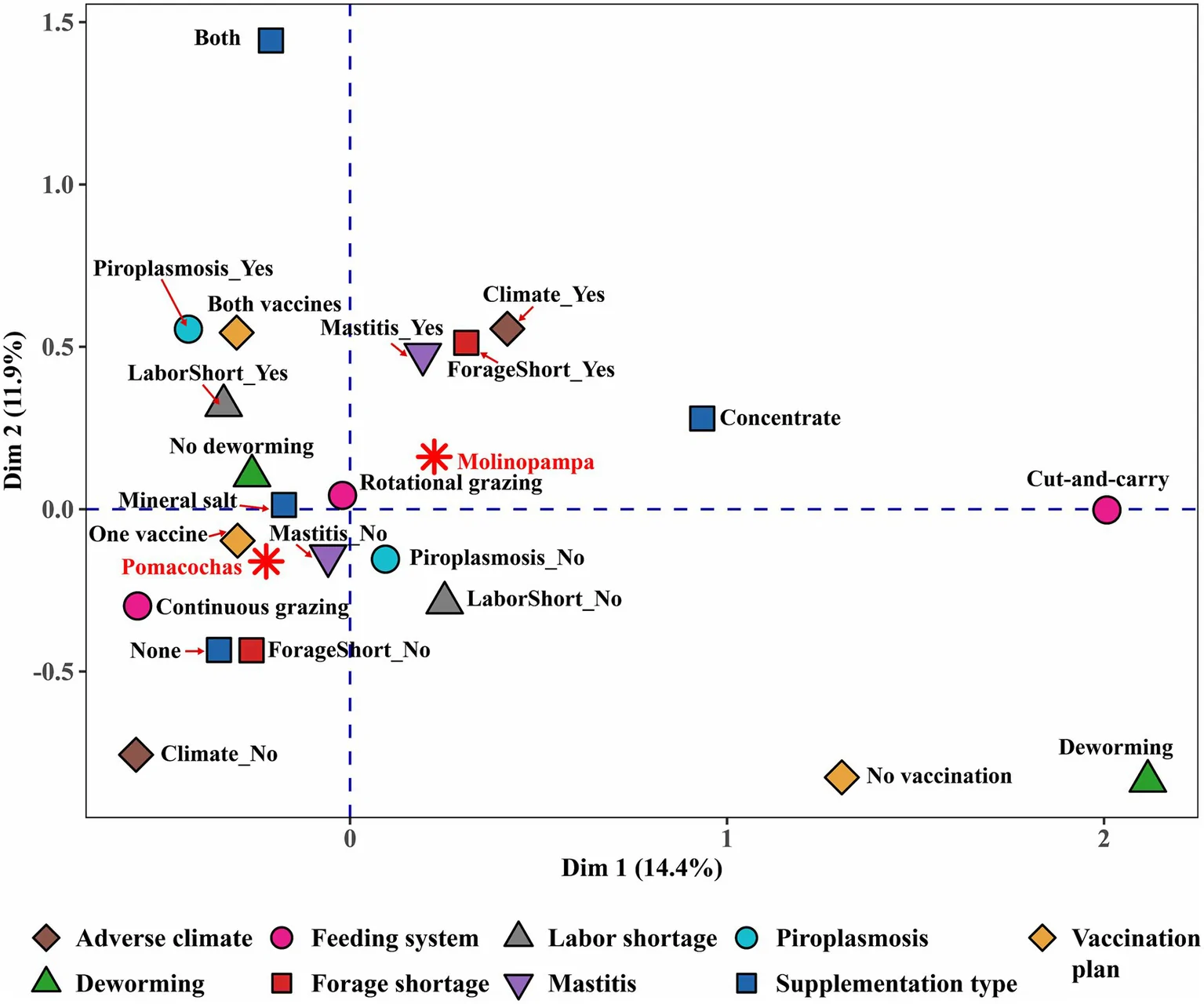
Multiple correspondence analysis of productive-health management and operational challenges.

#### 3.7.3 Economic-institutional linkages and commercial challenges

The multiple correspondence analysis of the six economic, institutional, and commercial variables (sales channel, perceived profitability, source of technical assistance, unstable market prices, high operating costs, and inadequate infrastructure) yielded a cumulative percentage of 26.9% in its first two dimensions (Dim. 1 = 15.8%; Dim. 2 = 11.1%; Figure 5) and showed a separation between the micro-watersheds based on the mode of commercialization rather than on operational challenges. Molinopampa was associated with the association or cooperative channel, the perception of non-profitability, and the challenge of unstable market prices, whereas Pomacochas was associated with the processing company channel, the perception of profitability, and sources of technical assistance such as SENASA, the municipality, and multiple sources. The distance between the two micro-watersheds was 0.700, the highest of the three analyses. In contrast, the challenges related to infrastructure and operating costs did not separate the micro-watersheds; rather, both shared them equally, confirming that these are common concerns across the entire region and not features that distinguish one micro-watershed from the other.

**Figure 5:**
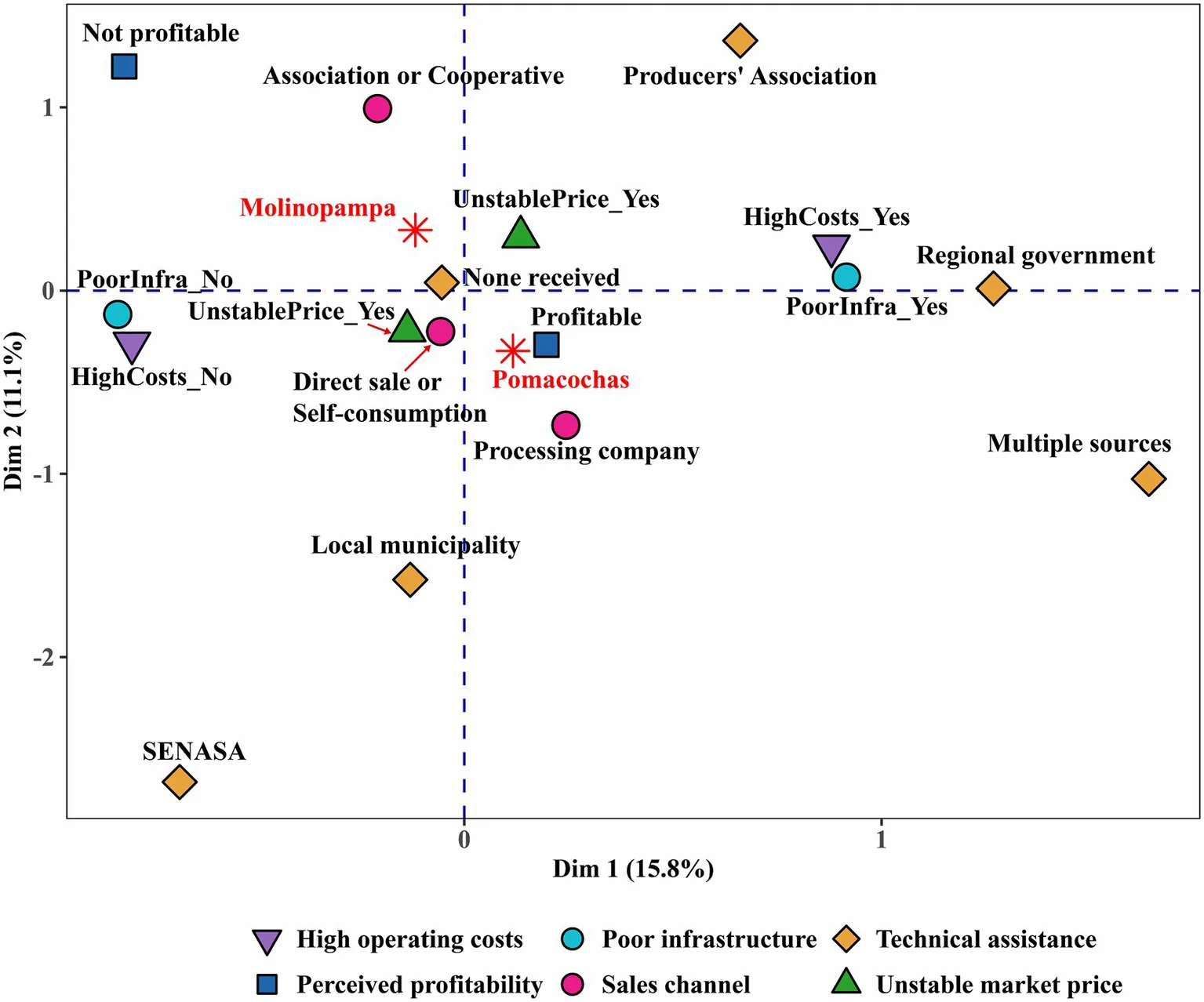
Multiple correspondence analysis of economic-institutional linkages and commercial challenges.

## 4 Discussion

In the present study, experience among livestock producers did not show a significant association with milk yield. This finding is consistent with that reported by Ahmed et al. (38), who noted that years of experience do not necessarily translate into improved practices adopted in dairy production systems (39); practices that are necessary to increase quality and production. In this regard, technical training constitutes a determining factor for herd size management and the implementation of adequate milking practices (40). Likewise, a higher proportion of women as producers was observed in Molinopampa compared with Pomacochas. This pattern could be related to the so-called feminization of agriculture, a process in which the male departure from small-scale agriculture translates into greater visibility of women as producers and farm managers (41). Rural male migration alters the division of labor on the farm and transfers management tasks and decisions to women, although structural inequalities may limit their effective decision-making authority (42). Within this framework, the greater female participation in Molinopampa may reflect a more advanced territorial dynamic of farm labor reorganization.

In addition, differences in educational level were observed between both micro-watersheds, with a higher proportion of producers without formal education in Molinopampa and with secondary education in Pomacochas; these values are consistent with the urban–rural educational gap documented in Peru (43). This gap has direct productive implications, as it has been documented that producers who reached the secondary level had 2.7 times more knowledge about veterinary inputs compared with those with primary education (44). These characteristics should be taken into account for technology transfer strategies.

The breed composition of the herds constituted one of the most marked contrasts between micro-watersheds. The Brown Swiss breed predominated in the herds of Molinopampa, whereas Simmental and Fleckvieh were more frequent in Pomacochas. Among breed preferences, it has been documented that dual-purpose systems have been traditionally preferred by small-scale producers due to their flexibility, their adaptation to climatic conditions, and their lower requirement for investment and technical support (45). However, dual-purpose systems usually show a greater preference for milk production, reserving the income from meat for periods of economic scarcity, but it must be considered that the productive orientation varies according to the producer and the local market (46). The predominance of Brown Swiss in Molinopampa is consistent with an orientation toward greater dairy specialization, although this breed is known to show greater mobilization of body reserves and a decrease in milk production in challenging high-altitude environments, compared with local dual-purpose breeds (47). The greater presence of Simmental and Fleckvieh in Pomacochas, breeds recognized for their dual meat and dairy aptitude, suggests a strategy oriented toward productive flexibility and rusticity.

This contrast was also reflected in the typology of the herds. Molinopampa presented a higher proportion of two-breed and multi-breed herds, whereas Pomacochas was characterized by single-breed herds, a pattern that the multiple correspondence analysis confirmed by associating greater breed diversity with Molinopampa. The combination of several breeds in the same herd can be interpreted as a strategy of productive diversification, although in Latin American dual-purpose systems, the definition of breed composition is often constrained by the scarcity of productive records (48).

Likewise, the number of lactating cows showed one of the most marked and statistically significant correlations with daily milk yield per herd. This finding is consistent with that reported by various studies, where lactating cows have been identified as one of the main determinants of total milk yield and can be quantified in the herd with an estimated increase of 0.25 L day^−1^ in milk yield per additional cow, depending on the breed (49, 50). In contrast, producers in Pomacochas reported a larger total area allocated to cattle raising. Total farm area showed a positive correlation with the number of lactating cows, which suggests that greater land availability is associated with a higher number of lactating cows per herd. In small-scale dairy systems, farm area and land allocated to grazing directly condition the herd size managed by the family unit and the possibility of growing their own forages; dairy producers with larger farm areas tend to allocate more land to grazing, maintain larger herds, and sustain systems that are less dependent on external inputs (51). The inverse mechanism is also documented, where the scarcity of land for grazing and forage production significantly reduces the milk yield potential in small-scale producers, even when comparing farms with similar animal health and housing conditions (52).

Taramuel-Taramuel et al. (53) showed that larger farms were associated with greater production per cow and with greater dependence of household income on dairy activity. Overall, the greater land availability in Pomacochas supports a higher capacity to sustain herds with more lactating cows. However, this territorial advantage requires being combined with adequate management practices.

Regarding animal health, clinical mastitis was significantly more frequent in Molinopampa, whereas piroplasmosis did not differ significantly between micro-watersheds. The higher self-reported occurrence of mastitis in Molinopampa should be interpreted cautiously, as the cross-sectional design does not allow the observed difference to be attributed to specific management or production factors (54). However, since these data correspond to self-reported perceptions by producers and not to confirmed clinical diagnoses, their interpretation should be made with caution. Notably, deworming was an infrequent practice in both micro-watersheds, with no significant differences between them. This low antiparasitic coverage constitutes a systemic limitation of the system, given that parasitism compromises the productive and reproductive performance of the herd and represents one of the main causes of economic losses in dairy production (55).

Molinopampa showed greater use of concentrate and dual vaccination, whereas Pomacochas recorded a higher milk price, a lower self-reported occurrence of clinical mastitis, and a greater orientation toward marketing through a local processing company. Gollin and Rogerson (56) showed that high transportation costs can generate differences in farm-gate prices between producers in remote areas and those with better access to urban markets. Differences in market accessibility could therefore be associated with the contrasting marketing patterns observed between Molinopampa and Pomacochas; however, geographical connectivity and distance to markets were not directly measured in this study. Similarly, Migose et al. (57) documented that localities with better accessibility to urban markets received USD 45.1/100 kg of milk compared with USD 32.8/100 kg in remote localities, representing a differential of 37.5%. In the present study, milk price represented the largest contrast between micro-watersheds, with a higher value in Pomacochas (1.67 vs. 1.52 PEN L^−1^). The observed price difference coincided with distinct marketing channels; however, the cross-sectional design does not allow determining whether market linkage or accessibility explains this difference.

The contrast in marketing channels represented one of the most notable findings of the study. Molinopampa primarily engaged with associations or cooperatives and direct sale or self-consumption, whereas Pomacochas was mostly integrated with a processing company. These two commercial models entail distinct advantages and limitations for the producer. Agricultural cooperatives play a relevant role in supporting small-scale producers by improving their access to the market, increasing their bargaining power, and facilitating access to inputs at better prices (53). However, in the Peruvian Andean context, milk marketing between small-scale producers and processors is usually developed without contracts that link the parties, which allows producers to change buyers according to the price and services offered, but also keeps them in a situation of low profitability (58). The higher perception of profitability observed in Pomacochas suggests that its integration with a formal processing company may be associated with more stable commercial conditions, although this type of integration usually requires higher quality and volume standards.

This pattern was confirmed by the multiple correspondence analysis, which located the cooperative channel and the perception of non-profitability on the side of Molinopampa, and the processing company channel and the perception of profitability on the side of Pomacochas.

Nevertheless, more than 87% of livestock producers in both micro-watersheds did not receive technical assistance. These results are consistent with the gaps that persist throughout the Amazonas Region, with approximately 87.06% of agricultural producers without technical assistance service (59). These figures are concerning, given that extension services constitute one of the main strategies to increase annual milk yield per cow, income from milk sales, and household income (60). Various studies report a positive association between access to extension services and dairy productivity, attributable to the better implementation of management practices transmitted by extension agents, as well as to the adoption of appropriate technologies, with a potential impact on the reduction of rural poverty (61, 62). The observed gaps highlight the need for stronger government intervention. The available evidence suggests that government intervention is positively associated with the adoption of climate-smart strategies aimed at reducing the operating costs of production units (63). This need is particularly pressing, given that livestock producers in both micro-watersheds identify high operating costs as a difficulty of dairy activity. In fact, the multiple correspondence analysis showed that both inadequate infrastructure and high operating costs did not differentiate the micro-watersheds, confirming that they constitute structural barriers shared at the regional level rather than problems specific to a particular locality.

The research had some limitations that should be considered for a more accurate interpretation of the results. The first concerns the cross-sectional design, which captured the systems at a single point in time and therefore did not allow for evaluating the temporal evolution of the observed differences or establishing causal relationships. In addition, the research relied on self-reported data from producers regarding disease occurrence and perceived challenges, which may have been influenced by recall and perception biases rather than reflecting confirmed clinical diagnoses or objective measurements. Finally, the greater geographical accessibility of Pomacochas was proposed as an explanatory hypothesis for the commercial and price differences observed, but it was not measured directly, so its role should be confirmed in future research incorporating variables such as road connectivity and distance to markets.

## 5 Conclusion

This study characterized and compared the dairy cattle production systems of two micro-watersheds in the Peruvian Andean Amazon, revealing that their differences are not uniformly distributed across the aspects evaluated. The greatest contrasts were concentrated in milk marketing, in the breed composition of the herds, and in the price received, whereas the structural and productive variables showed differences of lower magnitude. Among the quantitative variables, milk price was the only variable with a large effect size (Cliff’s *δ* = −0.488), representing the largest quantitative contrast between micro-watersheds. Molinopampa was characterized by a higher proportion of women producers, greater use of concentrate and dual vaccination, and a commercial orientation toward associations or cooperatives and direct sale or self-consumption, although with a lower milk price and lower perceived profitability. Pomacochas was distinguished by its integration with a processing company, a higher milk price, and higher perceived profitability. The higher self-reported occurrence of mastitis in Molinopampa represented a significant health difference, whereas piroplasmosis did not differ significantly between micro-watersheds.

Despite these differences, both micro-watersheds shared common limitations, particularly the limited coverage of technical assistance and the challenges of infrastructure and high operating costs, which did not distinguish one locality from the other. Overall, these findings show that two micro-watersheds belonging to the same region can present productive systems with relevant functional and commercial differences, which suggests the appropriateness of considering these particularities in the design of dairy development policies. Future research could complement this characterization with longitudinal studies that allow understanding the evolution of these systems.

## Data Availability

The raw data supporting the conclusions of this article will be made available by the authors, without undue reservation.
