# Supplementary material for: Characterization of dairy cattle production systems in two micro-watersheds of the Peruvian Amazonian Andes
Source: Front Vet Sci. 2026 Oct 14;13:1936252. doi: 10.3389/fvets.2026.1936252 (PMC13713265; doi:10.3389/fvets.2026.1936252)
Supplement: Supplementary file 1 [file Image_1.pdf]

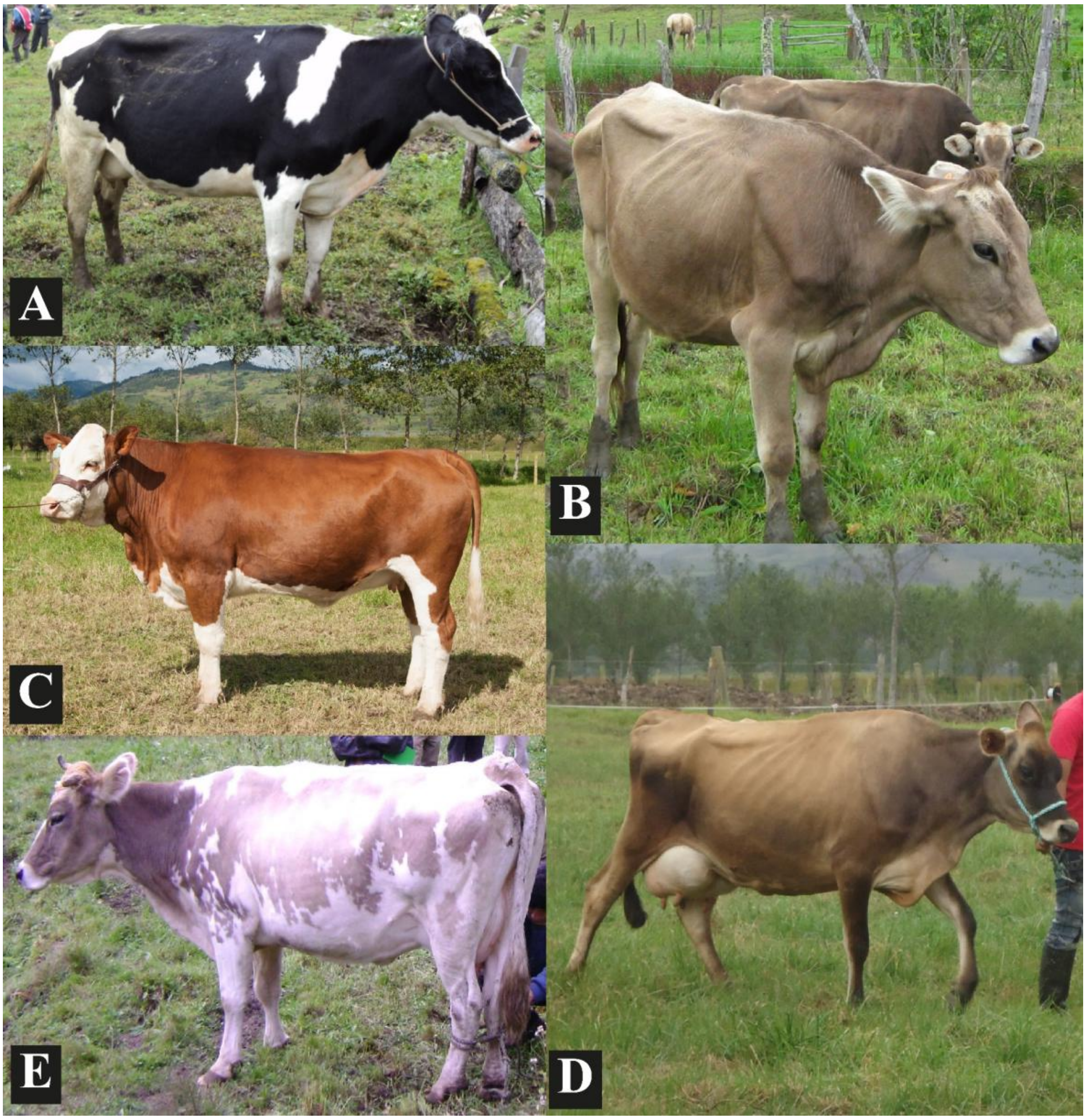

Figure S1. Representative photographs of the cattle breeds identified in the surveyed herds: (A) Holstein, (B) Brown Swiss, (C) Simmental–Fleckvieh, (D) Jersey, and (E) Criollo.
