# Supplementary material for: Characterization of dairy cattle production systems in two micro-watersheds of the Peruvian Amazonian Andes
Source: Front Vet Sci. 2026 Oct 14;13:1936252. doi: 10.3389/fvets.2026.1936252 (PMC13713265; doi:10.3389/fvets.2026.1936252)
Supplement: Supplementary file 2 [file Table_1.docx]

**Characterization of Dairy Cattle Production Systems in Two Micro-watersheds of the Peruvian Amazonian Andes**

Survey code: ___________	Micro-watershed: ( ) Molinopampa	( ) Pomacochas

Date: ____ / ____ / ______	Interviewer: __________________

**A. Sociodemographic profile**

**1. How many years of experience do you have as a livestock producer?**

Years: __________________

**2. Sex:**

( ) Female

( ) Male

**3. Educational level:**

( ) No formal education

( ) Primary

( ) Secondary

( ) Technical

( ) University

**B. Herd structure**

**4. How many lactating cows do you currently have in your herd?**

Number: __________________

**5. How many calves (male or female) do you currently have in your herd?**

Number: __________________

**6. How many breeding bulls do you currently have in your herd?**

Number: __________________

**C. Farm area and productive performance**

**7. What is the total area of your farm?**

Hectares (ha): __________________

**8. How many liters of milk does your herd produce per day?**

L day^−1^: __________________

**9. What is the selling price of milk?**

PEN L^−1^: __________________

**D. Breed composition of the herd**

**10. Which breeds are present in the herd? (You may select more than one option.)**

( ) Brown Swiss

( ) Fleckvieh

( ) Simmental

( ) Holstein

( ) Jersey

( ) Criollo

**11. Herd breed typology (classified by the interviewer based on the breeds reported above):**

( ) Single-breed

( ) Two-breed

( ) Multi-breed (three or more breeds)

**12. Which breeding method is used in your herd?**

( ) Natural mating

( ) Artificial insemination

( ) Both

**E. Productive and health management**

**13. Which of the following feeding systems is predominantly used for your herd?**

( ) Rotational grazing

( ) Continuous grazing

( ) Cut-and-carry

**14. What type of supplementation do you provide to your herd?**

( ) None

( ) Mineral salts only

( ) Concentrate only

( ) Mineral salts and concentrate

**15. Which vaccines are administered to your herd?**

( ) No vaccination

( ) One vaccine (against blackleg or rabies)

( ) Both vaccines (against blackleg and rabies)

**16. Do you deworm your cattle?**

( ) Yes

( ) No

**17. Has clinical mastitis occurred in the herd during the past year?**

( ) Yes

( ) No

**18. Has piroplasmosis occurred in the herd during the past year?**

( ) Yes

( ) No

**F. Economic and institutional linkages**

**19. What is the main channel through which you sell your milk?**

( ) Association or cooperative

( ) Processing company

( ) Direct sale or self-consumption

**20. How do you perceive the profitability of your livestock activity?**

( ) Profitable

( ) Not profitable

**21. What is the source of technical assistance for your farm?**

( ) Local municipality

( ) Regional government

( ) Association or cooperative

( ) SENASA

( ) Multiple sources

( ) None

**22. Do you have access to credit?**

( ) Yes

( ) No

**G. Challenges perceived by the producer**

**23. Indicate whether each of the following situations represents a challenge to your livestock activity:**

| Challenge | Yes | No |
| --- | --- | --- |
| Lack of pasture or forage | ( ) | ( ) |
| Adverse climatic conditions | ( ) | ( ) |
| Unstable market price | ( ) | ( ) |
| High operating costs | ( ) | ( ) |
| Inadequate infrastructure | ( ) | ( ) |
| Labor shortage | ( ) | ( ) |
